# Cutaneous *Mycobacterium haemophilum* Infection in an Immunosuppressed Host: A Case Report of Diagnostic Considerations and Microbiologic Confirmation

**DOI:** 10.1002/ccr3.73131

**Published:** 2026-07-22

**Authors:** Angus Lane, Amanda Godbolt, Sarah Morton, Heather Wiseby, Vivek Menon, Chris Coulter, Sushil Pandey, Andrew Burke

**Affiliations:** ^1^ Infectious Diseases Department The Prince Charles Hospital Herston Queensland Australia; ^2^ School of Clinical Medicine The University of Queensland St Lucia Queensland Australia; ^3^ Department of Dermatology The Royal Brisbane and Women's Hospital Herston Queensland Australia; ^4^ Rheumatology Department The Prince Charles Hospital Herston Queensland Australia; ^5^ Department of Anatomical Pathology Pathology Queensland Herston Queensland Australia; ^6^ Queensland Mycobacterium Reference Laboratory Pathology Queensland Herston Queensland Australia

**Keywords:** golimumab, immunocompromised host, *M. haemophilum*, nontuberculous mycobacteria, NTM, rheumatoid arthritis

## Abstract

Cutaneous *Mycobacterium haemophilum* infection may be missed due to its requirement for iron supplementation and low‐temperature incubation, making early consideration of NTM infection and use of molecular diagnostics essential, particularly in immunocompromised hosts where IRIS may mimic disease progression.

## 1 Introduction

*Mycobacterium haemophilum* is a rare, fastidious, slow‐growing nontuberculous mycobacterium requiring exogenous iron and low‐temperature incubation for growth [1, 2, 3]. These features contribute to frequent culture‐negative presentations and delayed diagnosis. Cutaneous disease typically affects cooler body sites such as extremities and ears [4]. Immunosuppressed hosts may demonstrate atypical histopathology with abundant acid‐fast bacilli but poorly formed granulomas [1, 5, 6]. We present a case of disseminated cutaneous *M. haemophilum* infection complicated by IRIS.

## 2 Case Presentation

**FIGURE 1:**
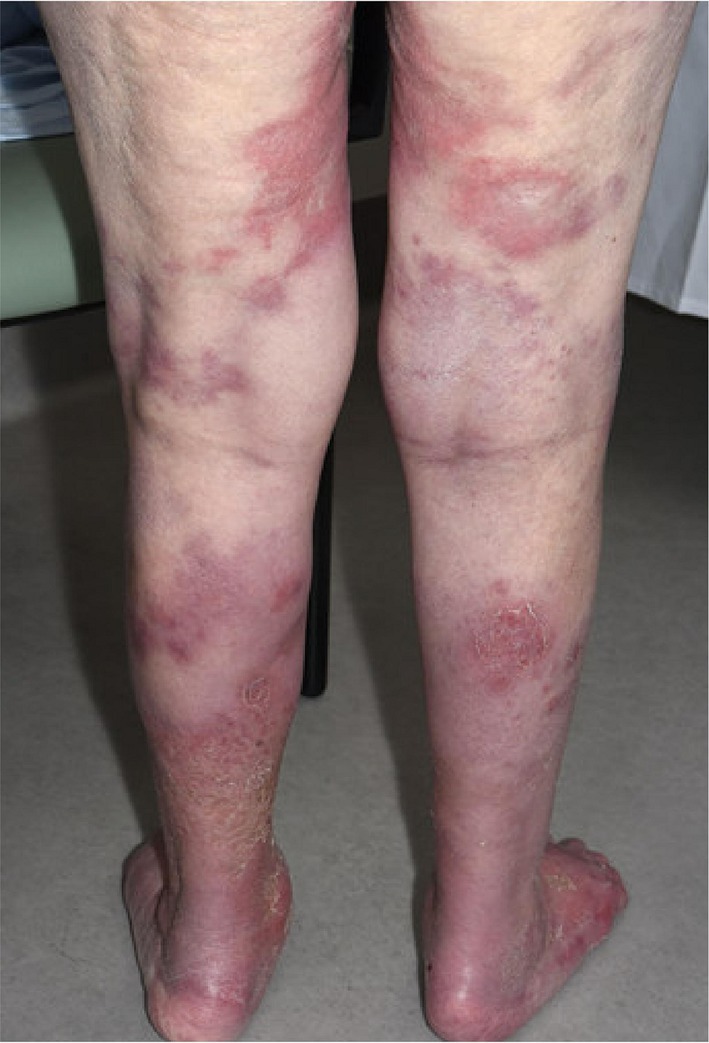
Erythematous indurated plaques and nodules with overlaying scale on posterior legs.

**FIGURE 2:**
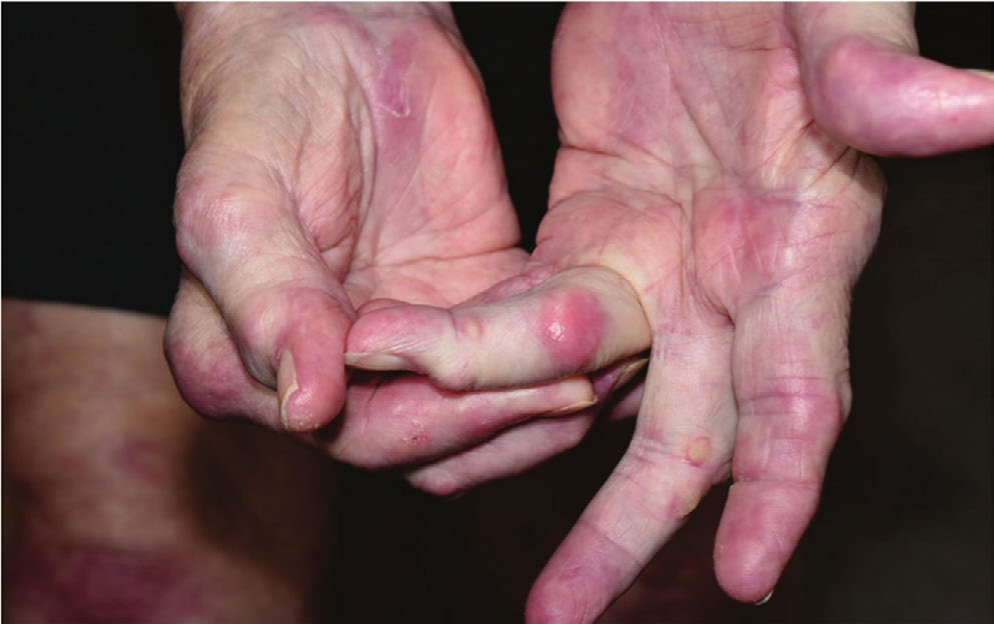
Indurated nodule left 4th finger.

A 61‐year‐old woman with seropositive rheumatoid arthritis (RA) treated with golimumab, methotrexate, and hydroxychloroquine presented in October 2024 with a several‐week history of progressively enlarging erythematous nodules and plaques affecting all four limbs and the right ear (Figures 1 and 2). She reported escalating arthralgia that significantly impaired hand function and mobility. There were no fevers, systemic symptoms, or recent travel.

**FIGURE 3:**
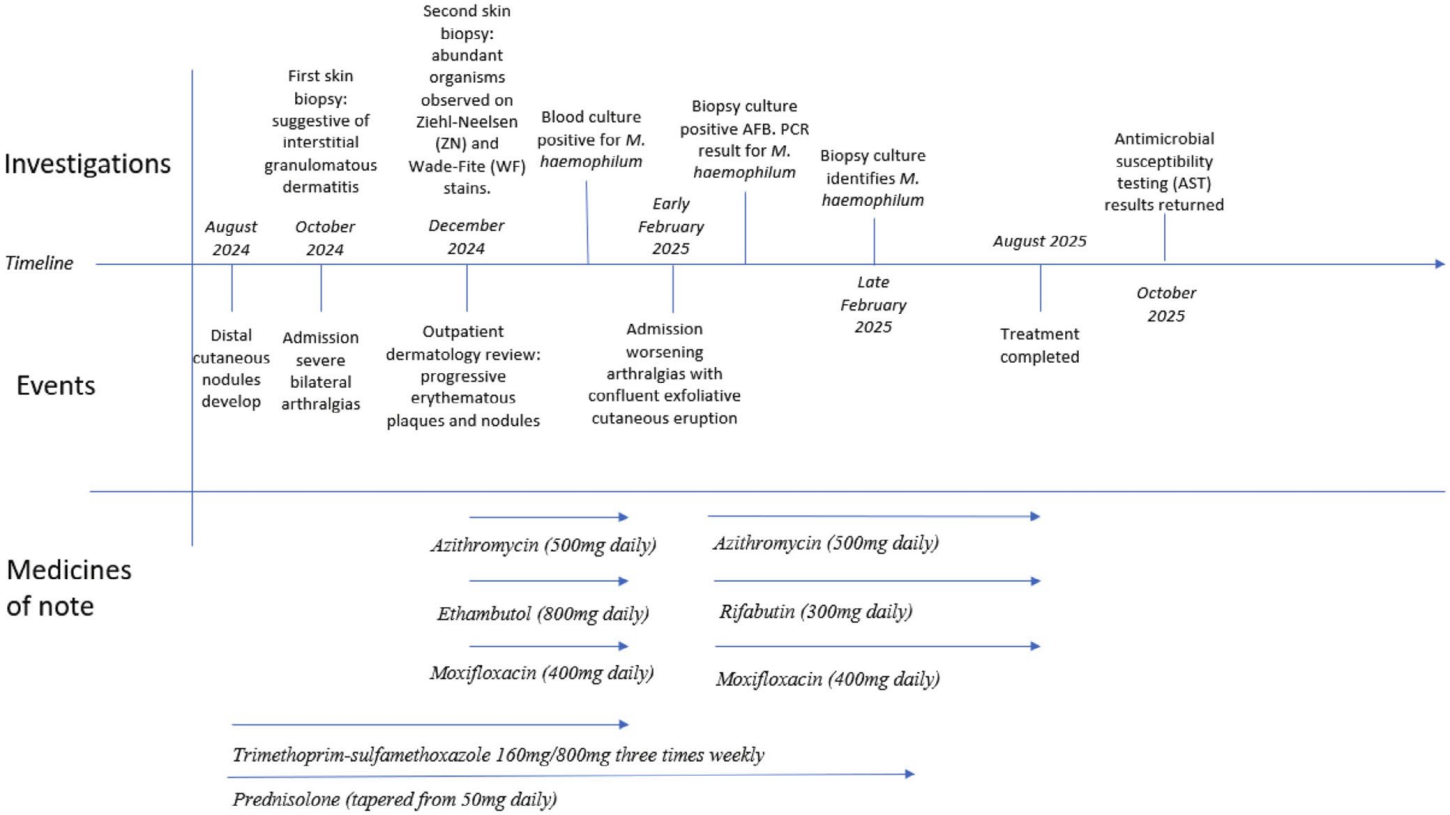
Clinical timeline.

**FIGURE 4:**
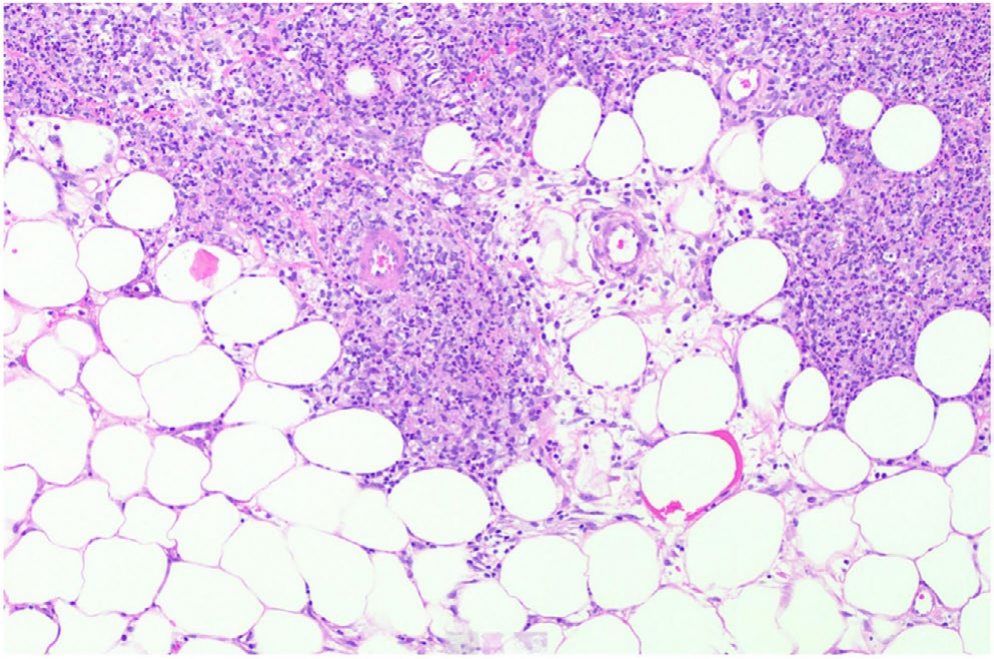
Numerous AFB at an area of inflammation in the dermo‐subcutaneous junction, a distribution typical of NTM infection and often missed on superficial biopsy.

**FIGURE 5:**
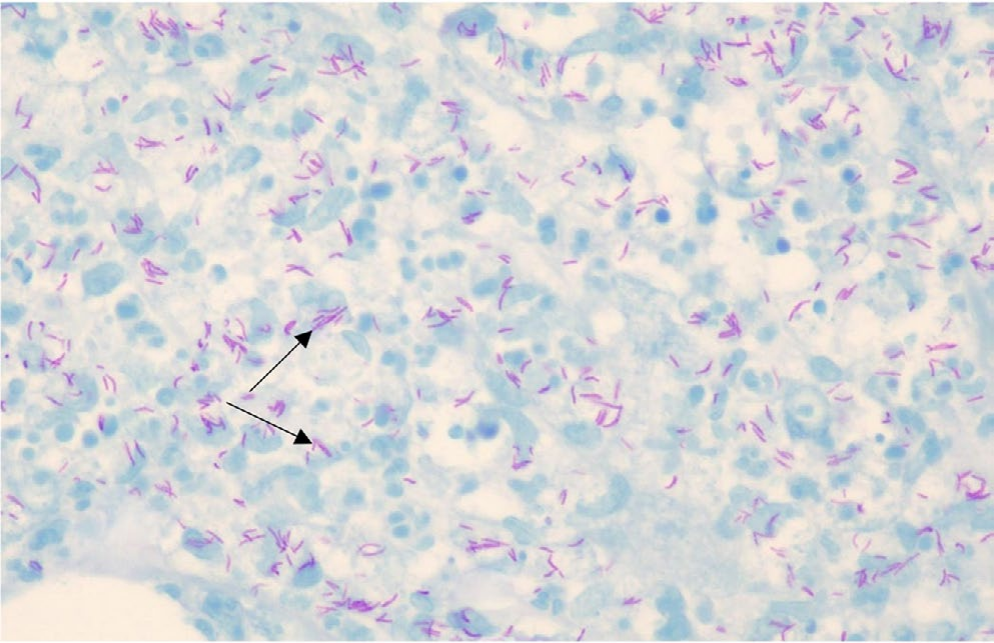
Numerous AFB 400× magnification ZN stain.

On examination, multiple firm nodules and plaques were noted on extensor surfaces of the arms and legs, with a prominent infiltrated lesion on the helix of the right ear. No lymphadenopathy was present. A superficial punch biopsy was initially thought to be consistent with interstitial granulomatous dermatitis, without organisms seen on histopathology. IGD was felt by rheumatology to be consistent with the clinical presentation. The patient was managed accordingly with increased immunosuppression including prednisolone (50 mg orally daily). Of note, this initial biopsy was taken to mid‐dermis, and did not include deep dermis or subcutis. Thus, the superficial nature of the initial biopsy resulted in mischaracterisation of the histiocytic infiltrate extending away from the deeper unsampled true pathological process. A Wade‐Fite (WF) stain was also performed; however, it was difficult to interpret as there was background reactivity in histiocytes. In the setting of improvement in skin changes following the initiation of prednisolone, a planned magnetic resonance imaging (MRI) scan and aspiration samples from the ankles were not pursued, and the patient was discharged on a tapering dose of prednisolone to 25 mg daily after a month. Trimethoprim‐sulfamethoxazole (160 mg/800 mg orally) was also prescribed three times a week for *Pneumocystis jirovecii* pneumonia (PJP) prophylaxis (Figure 3). Golimumab (100 mg) was recommenced. The patient was reviewed in dermatology clinic in December 2024 with worsening cutaneous lesions including progressive plaques and nodular disease. A second biopsy performed at this time revealed numerous acid‐fast bacilli on Ziehl–Neelsen staining (Figures 4 and 5).

## 3 Differential Diagnosis and Investigations

The initial biopsy was suggestive of interstitial granulomatous dermatitis. However, the presence of granulomatous inflammation prompted reconsideration of an underlying mycobacterial process, which had not been included in the initial differential. A repeat biopsy was therefore performed, with tissue specifically submitted for mycobacterial polymerase chain reaction (PCR) and culture. This demonstrated numerous acid‐fast bacilli on Ziehl–Neelsen staining (Figures 4 and 5). Growth was observed on chocolate agar and Löwenstein–Jensen (LJ) medium. Noting growth on LJ medium is an unusual finding for *Mycobacterium haemophilum* . Histopathology demonstrated a mixed neutrophilic and histiocytic infiltrate with granulomatous features, consistent with cutaneous mycobacterial infection. Targeted PCR for *M. leprae* was negative. Pan‐mycobacterial PCR identified *M. haemophilum* , and culture subsequently confirmed the organism. Blood culture was also positive for *M. haemophilum* at initial presentation, though this result would not be known for several weeks.

## 4 Results and Conclusion

Empirical therapy with azithromycin, ethambutol, and moxifloxacin was initiated and later changed to azithromycin, moxifloxacin, and rifabutin when *M. haemophilum* was confirmed. Treatment was complicated by a widespread exfoliative eruption consistent with a drug reaction possibly due to ethambutol and by clinical deterioration attributed to immune reconstitution inflammatory syndrome (IRIS). IRIS manifested approximately 8 weeks after commencement of antimycobacterial therapy, with paradoxical worsening of erythema, enlargement of existing lesions, and increased tenderness despite microbiological improvement. Antimicrobials were temporarily withheld and then reintroduced with corticosteroid support. The patient demonstrated improvement in cutaneous lesions and mobility over 12 weeks, and MRI excluded deeper involvement. Total treatment duration was 24 weeks before cessation. After completion of therapy, antimicrobial susceptibility testing (AST) results were returned from PathWest, the only Australian laboratory to offer this testing, which demonstrated resistance to rifampicin and doxycycline and sensitivity to amikacin, ciprofloxacin, clarithromycin, cotrimoxazole, linezolid, and minocycline.

## 5 Discussion

This case illustrates the diagnostic complexity of cutaneous *M. haemophilum* infection in an immunosuppressed patient with RA. The initial presentation mimicked rheumatologic skin disease, and early biopsy was superficial, missing deeper pathology. Repeat biopsy and targeted microbiologic testing were essential for diagnosis in this case. Routine AFB culture protocols frequently fail to recover *M. haemophilum* because they do not meet the organism's strict metabolic and environmental requirements. *M. haemophilum* depends on exogenous iron, typically supplied as hemin or ferric ammonium citrate, for growth, and this requirement is not fulfilled by standard mycobacterial media [1, 2]. As a result, cultures incubated without iron supplementation remain negative despite the presence of abundant organisms on smear [3]. Growth on chocolate agar reflects the availability of hemin within the medium, highlighting the organism's dependence on iron‐rich substrates [3]. The organism also grows optimally at 30–32 C rather than the 35°C–37°C used in routine mycobacterial workflows, further contributing to false‐negative culture results [1, 3]. These features underscore the importance of explicit laboratory notification when *M. haemophilum* is suspected so that modified culture conditions can be implemented. The organism's cutaneous tropism reflects these growth preferences, with lesions typically affecting the extremities and cooler body sites as was seen in our patient's pattern of lesions on the limbs and ears [4].

Routine culture methods frequently fail to recover *M. haemophilum* because they do not meet the organism's metabolic requirements. These features explain why the species typically does not grow on conventional Löwenstein–Jensen or Middlebrook media. In our case, however, growth occurred on Löwenstein–Jensen medium supplemented with pyruvate at both 32 C and 36 C, an observation that is unusual and not fully explained by standard growth characteristics. No iron supplementation is routinely added to this medium in our laboratory, and the basis for this growth remains unclear. Occasional recovery of *M. haemophilum* from MGIT has been reported, suggesting that strain‐level variation or unrecognized media components may permit growth under conditions generally considered non‐permissive [5].

In our case, the repeat biopsy demonstrated the characteristic pattern of a mixed neutrophilic and histiocytic infiltrate with poorly formed granulomas, a finding accentuated in immunosuppressed hosts [3, 6]. The granulomas themselves may include lymphocytes, monocytes, multinucleated giant cells and granulocytes [1]. Organisms are often concentrated at the dermo‐subcutaneous junction, a distribution that may be missed in superficial biopsies as occurred in the first biopsy samples in this case. The presence of abundant AFB on Ziehl–Neelsen, Wade–Fite or auramine stains should immediately prompt consideration of NTM infection and the need for modified culture conditions, particularly when routine AFB culture is negative. *M. haemophilum* often shows abundant AFB despite sparse granulomatous organization, especially in immunosuppressed hosts [1]. This may help distinguish it from *M. marinum* , which typically presents with more prominent granulomas and *M. ulcerans* , which tends to have fewer AFBs and appear more necrotising [1]. In the setting of granulomatous skin disease, other differentials for *M. haemophilum* include *M. chelonae* , *M. abscessus* , and *M. fortuitum* [1]. Early species‐level identification is essential given the variability in antimicrobial susceptibility across NTM species.

PCR expedited diagnosis in our case. Species‐level identification of *M. haemophilum* by pan‐mycobacterial PCR is reliable because the organism possesses distinctive 16S rRNA and hsp65 gene sequence signatures that clearly separate it from other slow‐growing NTM, enabling confident molecular confirmation even when culture is delayed or unsuccessful [1, 2]. This molecular approach bypasses the organism's fastidious growth requirements and can identify mycobacterial DNA directly from tissue, including specimens with low organism burden [1]. PCR is particularly useful when abundant AFB are seen histologically but routine culture conditions fail to support growth. Positive PCR needs to be interpreted with caution due to possible environmental contamination. PCR may reduce time to effective treatment given previously reported median time to culture positivity of 13 days on chocolate blood agar, 19 days in Mycobacteria Growth Indicator Tube (MGIT) broth, and 33 days on Middlebrook 7H10 agar [5].

Our case demonstrated clinical success with two active antibiotic agents of azithromycin and moxifloxacin. According to our AST testing the organism was resistant to rifampicin. Susceptibility testing for *M. haemophilum* remains challenging due to the absence of endorsed standardized methods [3, 7]. *M. haemophilum's* iron supplementation requirement interferes with standard in vitro susceptibility testing platforms, hindering testing and reporting [1, 3]. Disk agar elution has been proposed as a potential approach, but it is not validated, and reproducibility is uncertain [3]. Reported susceptibility patterns indicate consistent resistance to ethambutol, and variable activity of sulphonamides, while macrolides, fluoroquinolones, and rifamycins demonstrate the most reliable activity in clinical case series [5, 8, 9]. Prolonged combination therapy may be required for 12–24 months although optimal duration is unclear and shorter durations have been used, as in this case [7, 8].

Although TNF‐α inhibitors as a class are recognized risk factors for NTM infection, published cases have predominantly involved adalimumab, infliximab, or etanercept. In this context, our case expands the spectrum of biologic agents associated with *M. haemophilum* infection. Rather than implying a direct causal relationship with golimumab alone, this presentation likely reflects the cumulative immunosuppressive effect of TNF‐α blockade together with methotrexate [5, 7].

This case also highlights the challenge of managing IRIS, which may occur after initiating NTM therapy [10]. Our patient experienced a paradoxical worsening of skin lesions, consistent with IRIS, necessitating corticosteroid escalation and temporary cessation of antimicrobials 8 weeks into therapy. IRIS has been reported more frequently with *M. haemophilum* than other NTM species, though data remain limited [7, 10]. A previous case series reported 31% of their cohort being affected by IRIS, with a median duration between antimicrobial therapy and IRIS being 49 days [5]. The same case series identified microbiologic risk factors of a higher bacillary burden on initial histopathology and positive blood culture for *M. haemophilum* in conjunction with higher inflammatory markers at diagnosis, all criteria of which our patient met [5].

Strengths of this report include the expansion of the spectrum of TNF‐α inhibitors linked to *M. haemophilum* and practical insights into diagnosis and management of NTM in immunosuppressed hosts. Limitations include the inherent constraints of a single‐patient observation.

NTM infection should be considered in patients presenting with nodular skin lesions, particularly those who are immunocompromised. *M. haemophilum* remains diagnostically challenging due to its requirement for iron supplementation and low‐temperature incubation, leading to frequent false‐negative cultures; in this context, pan‐mycobacterial PCR can provide rapid, culture‐independent confirmation. Treatment may be complicated by immune reconstitution inflammatory syndrome, which can mimic disease progression. Notably, this case represents the first documented association between golimumab therapy and *M. haemophilum* , extending the recognized class‐wide vulnerability conferred by TNF‐α inhibition.

## 6 Patient Perspective

Little did I know this would be one of the hardest times of my life. Losing my independence was one of the worst things I've ever had to endure. Suffering from constant pain all over my body, 24 h a day, 7 days a week was unbearable. I would use my pillow to snuffle my crying while trying to fall asleep, hoping the new day would bring relief from all I was going through. Pain relief and heat packs only gave short‐term comfort. I developed a rash from the top of my head to the soles of my feet. I was using steroid cream several times a day but it only got worse. Turned out I was allergic. So I had to start again, trying a new cream. I didn't know what could or would come next!

The unknown can be very frustrating.

My hands and feet were so swollen and sore I was unable to feed myself or to walk for days at a time. I knew I wasn't safe to be at home on my own. Being in hospital getting the support and care from all levels was the best place to be. When I was struggling, there always was someone saying, let me help you with that.

Wow, what a relief it was to hear these two words, it's a “Bacterial Infection.”

This lifted my spirits. I was like, hey, we can get the treatment happening, I'm ready to take this on.

After being readmitted into hospital and working out what strain it was, I started antibiotics for the next several months. Everything started improving. I know this isn't over yet but life is starting to look brighter 1 day at a time. I couldn't be happier. If this publication helps at least one person to not go through what I have, my journey will have been worth it. Everything happens for a reason.

## Author Contributions

**Sushil Pandey:** writing – review and editing. **Vivek Menon:** writing – review and editing. **Sarah Morton:** writing – review and editing. **Heather Wiseby:** writing – review and editing. **Angus Lane:** conceptualization, investigation, writing – original draft, methodology, writing – review and editing, project administration. **Andrew Burke:** writing – review and editing, supervision, resources. **Amanda Godbolt:** writing – review and editing. **Chris Coulter:** writing – review and editing.

## Funding

The authors have nothing to report.

## Disclosure

No generative artificial intelligence tools were used in the conception, design, or writing of this manuscript.

## Consent

Written informed consent was obtained from the patient for publication of this case report and accompanying images.

## Conflicts of Interest

The authors declare no conflicts of interest.

## Data Availability

Research data are not shared.
